# Polarity inversion reorganizes the stem cell compartment of the trophoblast lineage

**DOI:** 10.1016/j.celrep.2023.112313

**Published:** 2023-03-28

**Authors:** Hatice O. Ozguldez, Niraimathi Govindasamy, Rui Fan, Hongyan Long, Karina Mildner, Dagmar Zeuschner, Britta Trappmann, Adrian Ranga, Ivan Bedzhov

**Affiliations:** 1 Embryonic Self-Organization Research Group, Max Planck Institute for Molecular Biomedicine, Röntgenstraße 20, 48149 Münster, Germany; 2 Bioactive Materials Laboratory, Max Planck Institute for Molecular Biomedicine, Röntgenstraße 20, 48149 Münster, Germany; 3 Electron Microscopy Facility, Max Planck Institute for Molecular Biomedicine, Röntgenstraße 20, 48149 Münster, Germany; 4 Laboratory of Bioengineering and Morphogenesis, Department of Mechanical Engineering, KU Leuven, Leuven, Belgium

## Abstract

The extra-embryonic tissues that form the placenta originate from a small population of trophectoderm cells with stem cell properties, positioned at the embryonic pole of the mouse blastocyst. During the implantation stages, the polar trophectoderm rapidly proliferates and transforms into extra-embryonic ectoderm. The current model of trophoblast morphogenesis suggests that tissue folding reshapes the trophoblast during the blastocyst to egg cylinder transition. Instead of through folding, here we found that the tissue scale architecture of the stem cell compartment of the trophoblast lineage is reorganized via inversion of the epithelial polarity axis. Our findings show the developmental significance of polarity inversion and provide a framework for the morphogenetic transitions in the peri-implantation trophoblast.

## Graphical abstract

**Graphical abstract:**
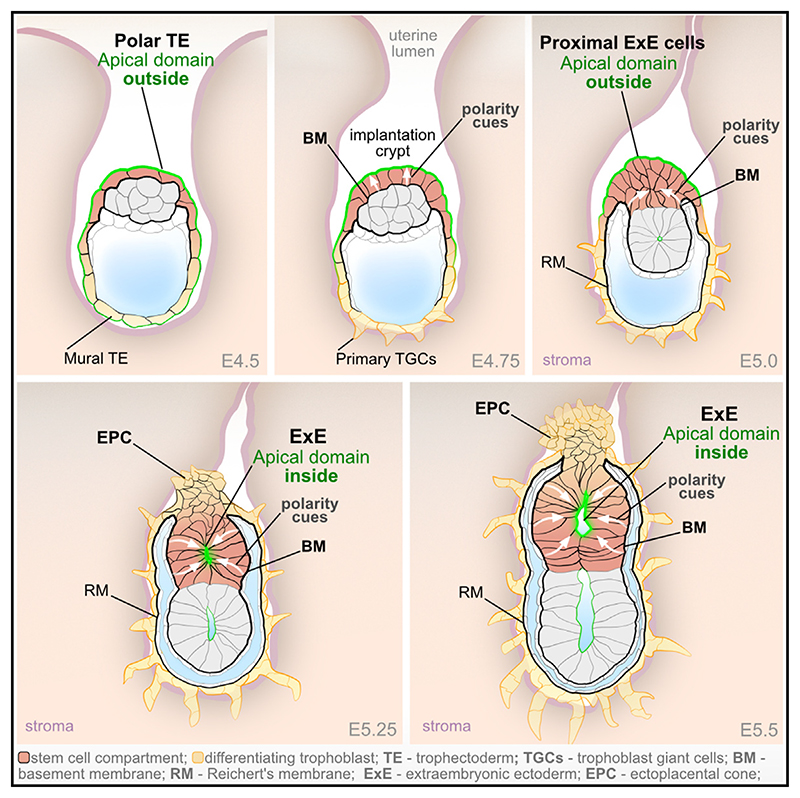

## Introduction

In mammals, intra-uterine embryonic development requires maternal input to support the bioenergetic requirements of the growing conceptus. This function is mediated by the placenta, a specialized organ that enables the exchange of nutrients, gas, and waste at the embryo-maternal interface. The stem cell founders of the placental tissues are specified during early embryogenesis and, although the signaling events involved in their cell fate specification have been extensively studied, the morphogenetic mechanisms that reshape the tissue architecture of the stem cell compartment are still poorly understood.

In the mouse, the placental lineage (trophoblast) originates from the outer blastomeres of the 16-cell stage morula. These cells establish defined apical and basolateral membrane domains, forming the first polarized epithelium during mouse embryogenesis. At the 32-cell stage, a central cavity (blastocoel) is formed, transforming the compacted morula into an early blastocyst.^1^ The inner cell mass (ICM) is positioned on one side of the cavity, which defines two trophectodermal (TE) compartments—the polar TE, in direct contact with the ICM and the mural TE, surrounding the blastocoel.^2^

The process of implantation is initiated at embryonic day 4.5 (E4.5), as the mural TE adheres to the surface of the uterus and differentiates into primary trophoblast giant cells (TGCs). In contrast with the terminal differentiation fate of the mural TE, the polar TE maintains its stemness and during the peri-implantation stages, between E4.5 and E5.0, forms the extra-embryonic ectoderm (ExE). The ExE is a pool of multipotent trophoblast progenitors, serving as founder cells of the placental tissues.^3^ Between E5.0 and E5.5, the proximal ExE cells give rise to the ectoplacental cone (EPC) cells, which establishes a second site of direct contact with the maternal environment, in addition to the primary TGCs.^4,5^

During early development, finely tuned cross-talk between the ExE and the epiblast, mediated by multiple signaling feedback loops, ensures proper development of the conceptus. For instance, the epiblast produces Fgf4, which binds to the Fgfr2 in the ExE and activates the downstream signaling cascade, maintaining the stem cell properties of the ExE. In turn, the ExE secretes endoproteases, which proteolytically process the Nodal precursor protein, produced in the epiblast.^6^ The confined space of the lumen (pro-amniotic cavity) acts as a micro-environment for Nodal processing, where the endoproteases and Nodal are released and accumulate.^7,8^

The cell fate transitions in the trophoblast during the peri-implantation stages are also associated with major changes in cellular morphology. At the blastocyst stage, the TE is shaped as a multicellular epithelial vesicle. The trophoblast cells that undergo differentiation to establish direct contact with the maternal tissues, such as the TGCs, lose their epithelial phenotype.^9^ In contrast, the trophoblast cells with stem cell properties, namely the E4.5 polar TE, the E5.5 ExE, as well as the trophoblast stem cells (TSCs) in culture, maintain their epithelial organisation.^3,10,11^ However, the orientation of the apical-basal axis in the E4.5 polar TE and the E5.5 ExE differs substantially. The apical domain of the polar TE faces the outside environment, whereas the apical domains of the ExE cells are clustered inside, surrounding the lumen within the core of the trophoblast.^11^

Clearly, the epithelial architecture of these tissues is reorganized during peri-implantation embryogenesis. The current model to describe the trophoblast morphogenesis, proposed by Perea-Gomez et al.,^12^ suggests that, at the early egg cylinder stage, the ExE forms a well-defined fold toward the epiblast. Thus, the apical cell membranes initially facing the outside environment are repositioned, establishing the inner surface of the fold. This elegant model has recently been put forward again and further emphasized by Christodoulou et al. (2018), Christodoulou et al. (2019), and Weberling and Zernicka-Goetz (2021),^13–15^ but the formation of such a structure may present several potential challenges for the organization and function of the ExE. As the fold is opened toward the maternal environment, the secreted ligands and endoproteases produced by the ExE could be released into the maternal fluids. This may affect the finely tuned signaling cross-talk between the trophoblast and the embryonic lineage. How the EPC, which is a solid mass of cells, arises from the ExE fold is also elusive. It is suggested that the proximal open end of the fold subsequently closes, but adhesive molecules are not expressed on the opposing surfaces; in contrast, anti-adhesive factors are deposited on the apical membranes.^11^

Taking the current model into account, here we examined the morphogenesis of the polar TE/ExE during peri-implantation embryogenesis. We found that, instead of through folding, the tissue-scale architecture of the trophoblast is globally reorganized via inversion of the apical-basal polarity axis.

## Results

### Reorganization of the epithelial polarity in the polar TE/ExE during implantation

**Figure 1:**
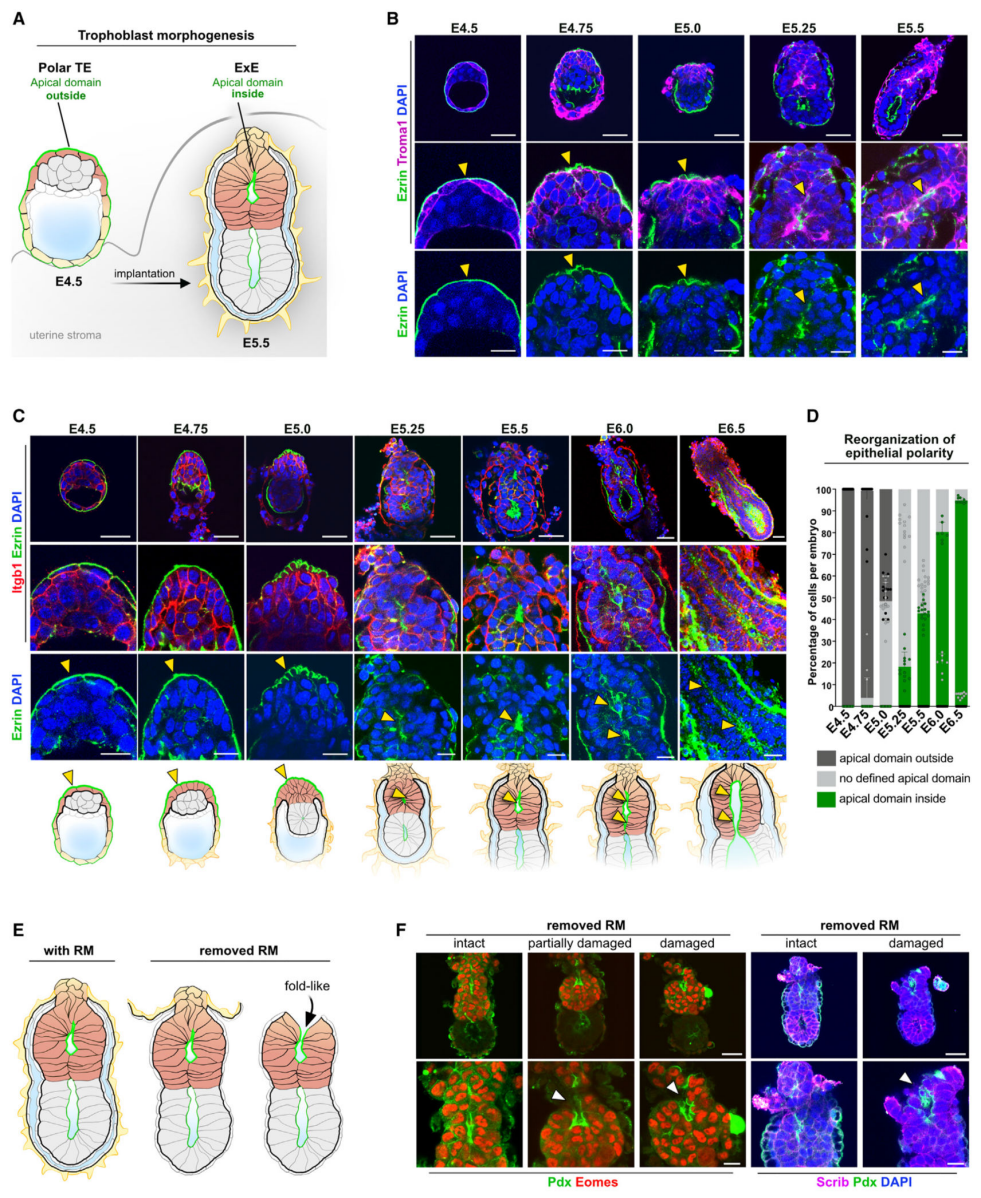
Reorganization of epithelial polarity in the polar TE/ExE (A) Schematic representation of the E4.5 to E5.5 transition and the reorganization of the polarity axis. (B) Representative images of E4.5, E4.75, E5.0, E5.25, and E5.5 embryos stained for Troma1, Ezrin, and DAPI. Arrowheads in the high-magnification images (middle and bottom) indicate the apical domain. (C) Representative images of E4.5, E4.75, E5.0, E5.25, E5.5, E6.0, and E6.5 embryos stained for Ezrin, integrin beta-1 and DAPI. Arrowheads in the high-magnification images (middle and bottom) indicate the apical domain. (D) Position of the apical domain of the polarTE and ExE cellsquantified based on Ezrin staining in E4.5, n = 25; E4.75, n = 16; E5.0, n = 18; E5.25, n = 16; E5.5, n = 19; E6.0, n = 7; and E6.5, n = 10 embryos. Data represent mean ± SD, at least three independent experiments. (E) Schematic representation of isolated E5.5 embryos with RM, removed RM (intact and damaged ExE). (F) Representative images of E5.5 embryos with intact and damaged ExE after RM removal. The embryos are stained for Eomes, Pdx (left) or Scrib, Pdx and DAPI (right). Arrowheads indicate damaged ExE. Scale bars B, C, F = 50 μm (low magnification) and 20 μm (high magnification). See also Figure S1.

During implantation, the primary TGCs establish the first contact between the embryo and the maternal tissues, followed by a second site of embryo-maternal interactions mediated by the cells of the EPC.^4,5,9^ As the implanting embryo is concealed by the decidua (Figure 1A), the recovery of peri- and early post-implantation embryos requires manual dissection of the implantation site. In addition, conventional immunohistochemistry protocols require the removal of the Reichert’s membrane (RM), a thick layer of extracellular matrix (ECM) proteins sandwiched between the parietal endoderm and the TGCs.^16^ As the early embryos are relatively small and particularly fragile, the process of disconnecting the conceptus from the maternal tissues and the manual removal of the RM can easily compromise the integrity of the egg cylinder. Therefore, to minimize the chance of damaging the concepti, we examined the organization of the polar TE/ExE in embryos with RM.

We isolated E4.5, E4.75, E5.0, E5.25, E5.5, and E6.0/E6.5 embryos and stained them for the apical marker Ezrin, in combination with the trophoblast marker cytokeratin 8 (Troma1) or the basolateral maker integrin beta-1. We found that the apical domain of the E4.5–E4.75 polar TE cells and the E5.0 proximal trophoblast cells face to the outside environment. Between E5.0 and E5.5, the Ezrin signal was detectable in the core of the ExE, which also contained cells without a defined apical domain, indicating an ongoing process of a reorganization of the apical-basal polarity (Figures 1B–1D). Staining for the anti-adhesive factor podocalyxin (Pdx) confirmed the establishment of the apical membrane identity in the E5.25–E5.5 ExE (Figure S1A). By E6.5, the tissue-scale reorganization of the epithelial polarity in the trophoblast was completed and a central (pro-amniotic) cavity surrounded by apical membranes spanned through both the ExE and the epiblast compartments of the egg cylinder (Figures 1C and 1D).

Interestingly, we did not observe ExE folding while using intact embryos for analysis. A closer look at the published representative images of Perea-Gomez et al.^12^ revealed that the RM was removed from the embryos used in that study, and the proximal part of the ExE displayed signs of mechanical damage and cell debris. The RM was also removed in the more recent reports.^13–15^ As the RM connects the basement membrane (BM) of the egg cylinder at the proximal site of the ExE (Figure 1E), this site is the most prone to mechanical damage upon removal of the RM. Indeed, dissecting the RM has often compromised the integrity of the E5.5 ExE (marked by Eomes expression) in our own samples, exposing apical membrane domains to the outside environment in a fold-like structure (Figures 1E and 1F). At an earlier stage (E5.0), disconnecting the embryo from the implantation site resulted in the collapse of the blastocoel, shrinking the trophoblast tissues, which were also prone to mechanical damage upon dissection of the mural TE/TGCs compartment (Figures S1B and S1C). Thus, preserving the integrity of the conceptus is critical for examining the process of trophoblast morphogenesis.

**Figure 2:**
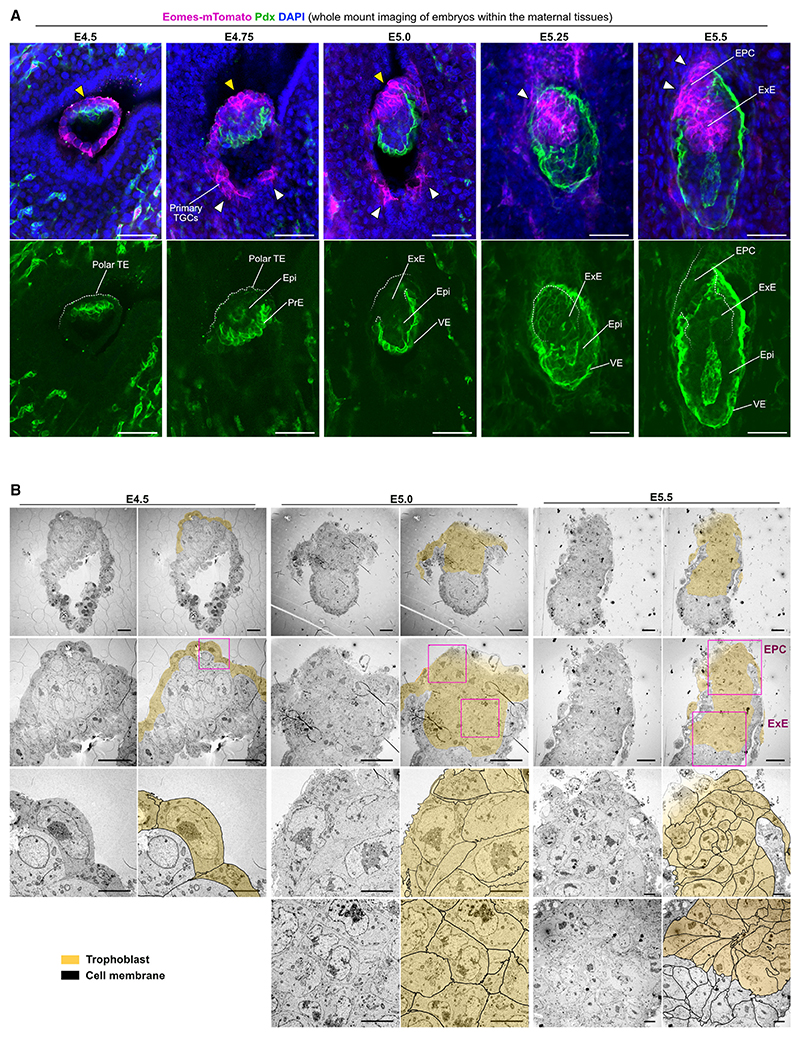
Trophoblast morphogenesis in the implanting embryo (A) Representative images of whole-mount staining for mTom, Pdx, and DAPI of uterine tissues containing E4.5, E4.74, E5.0, E5.25, and E5.5 Eomes-mTom embryos. White arrowheads indicate the formation of the embryo-maternal interface, yellow arrowheads indicate the E4.5, E4.75 polar TE, and posterior trophoblast cells of the E5.0 ExE. (B) Representative images of transmission electron microscopy images of E4.5, E5.0, and E5.5 embryos. The color overlay marks the trophoblast (yellow) and the cell membranes of the trophoblast cells (black). Scale bars (A), 50 μm; (B), 20 μm (top two panels), 5 μm (bottom two panels). See also Figure S2.

To examine the polar TE/ExE morphogenesis when preserving the integrity of both the conceptus and the implantation site, we performed whole-mount tissue clearing and imaging of embryos within the maternal tissues. We used reporter embryos expressing membrane-bound tdTomato (mTom) under the control of Eomes regulatory sequences (Eomes-mTom knock-in).^17^ The uterine tissues containing E4.5, E4.75, E5.0, E5.25, E5.5, E6.0, and E6.5 Eomes-mTom embryos were subjected to a tissueclearing procedure, rendering the samples completely transparent. A three-dimensional (3D) imaging analysis revealed the establishment of the first embryo-maternal interactions mediated by the (distal) mural TE/primary TGCs between E4.5 and E5.0 (Figure 2A, E4.5, E4.75, and E5.0, white arrowheads, Videos S1, S2, and S3). At this time, the proximal trophoblast (E4.5/E4.75 polar TE and E5.0 ExE) is still not in direct contact with the maternal tissues (Figure 2A, yellow arrowheads, Videos S1, S2, and S3). Later, at E5.25–E5.5, a second embryo-maternal interface is established at the proximal trophoblast (Figure 2A, E5.25 and E5.5, white arrowheads, EPC). Meanwhile, the ExE continuously proliferates (E5.25–E6.5), expanding the early egg cylinder (Figures 2A and S2, Videos S4, S5, S6, and S7). Importantly, these progressive changes in the morphology of the trophoblast lineage were not associated with the establishment of a fold in the polar TE/ExE.

### Cell proliferation and morphogenesis of the polar TE/ExE

During the pre-implantation stage, the zygote undergoes cleavage divisions that generate progressively smaller cells. Upon implantation, extensive cell proliferation rapidly generates cell mass, expanding the embryonic and the extra-embryonic line-ages.^18^ Thus, cell proliferation is a key aspect of the morphogenetic program in the post-implantation conceptus. Therefore, we examined the growth and the associated changes in the cell shape of the polar TE/ExE during the E4.5–E5.5 transition.

**Figure 3:**
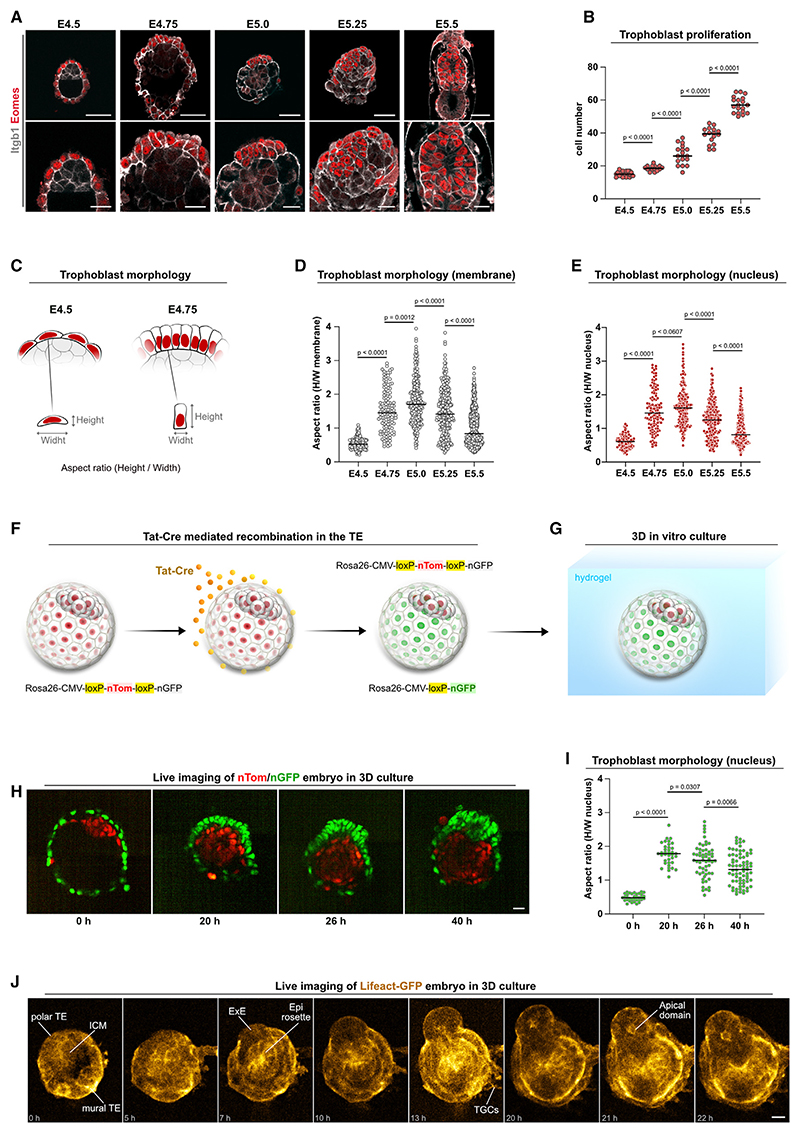
Cell proliferation and morphogenesis of the polar TE/ExE (A) Representative images of E4.5, E4.75, E5.0, E5.25, and E5.5 embryos stained for Eomes and integrin beta-1. (B) Number of polar TE ExE cells in E4.5, n = 25; E4.75, n = 16; E5.0, n = 18; E5.25, n = 16; and E5.5, n = 19 embryos based on Eomes staining from. Data represent mean value, two-sided unpaired Student’s t test, at least three independent experiments. (C) Schematic representation of the changes in the trophoblast cell morphology. (D) Height/width aspect ratio of the cell membrane based on integrin beta-1 staining at E4.5 (n = 180 cells in 25 embryos), E4.75 (n = 123 cells in 16 embryos), E5.0 (n = 242 cells in 18 embryos), E5.25 (n = 305 cells in 16 embryos), and E5.5 (n = 524 cells in 19 embryos). Data represent mean value, two-sided unpaired Student’s t test, at least three independent experiments. (E) Height/width aspect ratio of the nucleus based on Eomes staining at E4.5 (n = 173 cells in 24 embryos), E4.75 (n = 116 cells in 15 embryos), E5.0 (n = 218cellsin 17 embryos), E5.25 (n = 265 cells in 14 embryos), and E5.5 (n = 518 cells in 19 embryos). Data represent mean value, two-sided unpaired Student’s t test, at least three independent experiments. (F) Schematic representation of the Tat-Cre-mediated recombination in the TE of mTom/nGFP embryos. (G) Schematic representation of mTom/nGFP embryo culture in polyethylene glycol (PEG) hydrogel. (H) Representative images of time-lapse recording of Tat-Cre-treated mTom/nGFP embryo in PEG hydrogel. (I) Height/width aspect ratio of the nucleus in the polar TE/ExE based on nGFP signal (0 h, n = 34; 20 h, n = 37; 26 h n = 58; 40 h, n = 75 cells). Data represent mean value, three independent experiments, two-sided unpaired Student’s t test. (J) Representative images of time-lapse recording of Lifeact-GFP embryo in DexMA hydrogel. Scale bars (A), 50 μm (top) and 20 μm (bottom); (H) and (J), 20 μm.

First, we performed an ultrastructural analysis of E4.5, E5.0, and E5.5 embryos using transmission electron microscopy, which revealed the monolayer morphology of the E4.5 polar TE forming the nascent ExE at E5.0. Between E5.0 and E5.5, the trophoblast expanded further in both distal (ExE) and proximal (EPC) directions (Figure 2B). To quantify the increase of cell number in the polar TE/ExE and the associated changes in the cell shape, we stained E4.5, E4.75, E5.0, E5.25, and E5.5 embryos for Eomes and integrin beta-1 (Figure 3A). We observed an approximately 3-fold increase in cell number within the E4.5–E5.5 developmental window (Figure 3B). Between E4.5 and E5.0, the polar TE cells formed a tightly packed cohort, temporarily increasing their membrane and nuclear height-to-width ratio (Figures 3C–3E).

To capture the dynamics of this process, we performed live imaging using double-fluorescent reporter embryos expressing nuclear-targeted tdTomato (nTom) flanked by *LoxP* sites, followed by nuclear-targeted GFP (nGFP).^19^ We labeled the trophoblast cells by treating embryos at the blastocyst stage with cell-permeant Cre protein (Tat-Cre). This approach results in Tat-Cre uptake only by the directly exposed TE cells, whereas the ICM remains out of reach.^20,21^ Thus, the nTom cassette is excised only in the trophoblast, which gains a constitutive nGFP expression (Figure 3F). We performed time-lapse microscopy on nTom/nGFP embryos cultured *in vitro* using our recently established 3D biomimetic culture system^9^ (Figures 3G and 3H, Video S8). Similar to snapshot observations of embryos recovered from the uterus, we found that the increasing cell number results in crowding of the trophoblast cells at the proximal region, which is associated with an increase in the nuclear height to width aspect ratio (Figures 3H and 3I). In addition, we cultured embryos expressing the actin cytoskeleton reporter Lifeact-GFP.^22^ Time-lapse imaging revealed the formation of a polarized epiblast rosette, alongside the expansion of the polar TE/ExE, followed by the establishment of an apical domain in the trophoblast, marked by the accumulation of cortical actin in the center of the ExE (Figure 3J and Video S9).

It is worth mentioning that the 3D *in vitro* culture approach is a reductionist approximation of the maternal environment and, therefore, it lacks the tissue complexity of the implantation niche. Although embryos cultured *in vitro* exhibit a slower developmental pace and some perturbations in the overall embryo shape, our observations are in line with the results of the whole-mount imaging *in situ* (Figure 2A), revealing no signs of polar TE/ExE tissue folding.

### Inversion of the apical-basal polarity axis in the trophoblast

**Figure 4:**
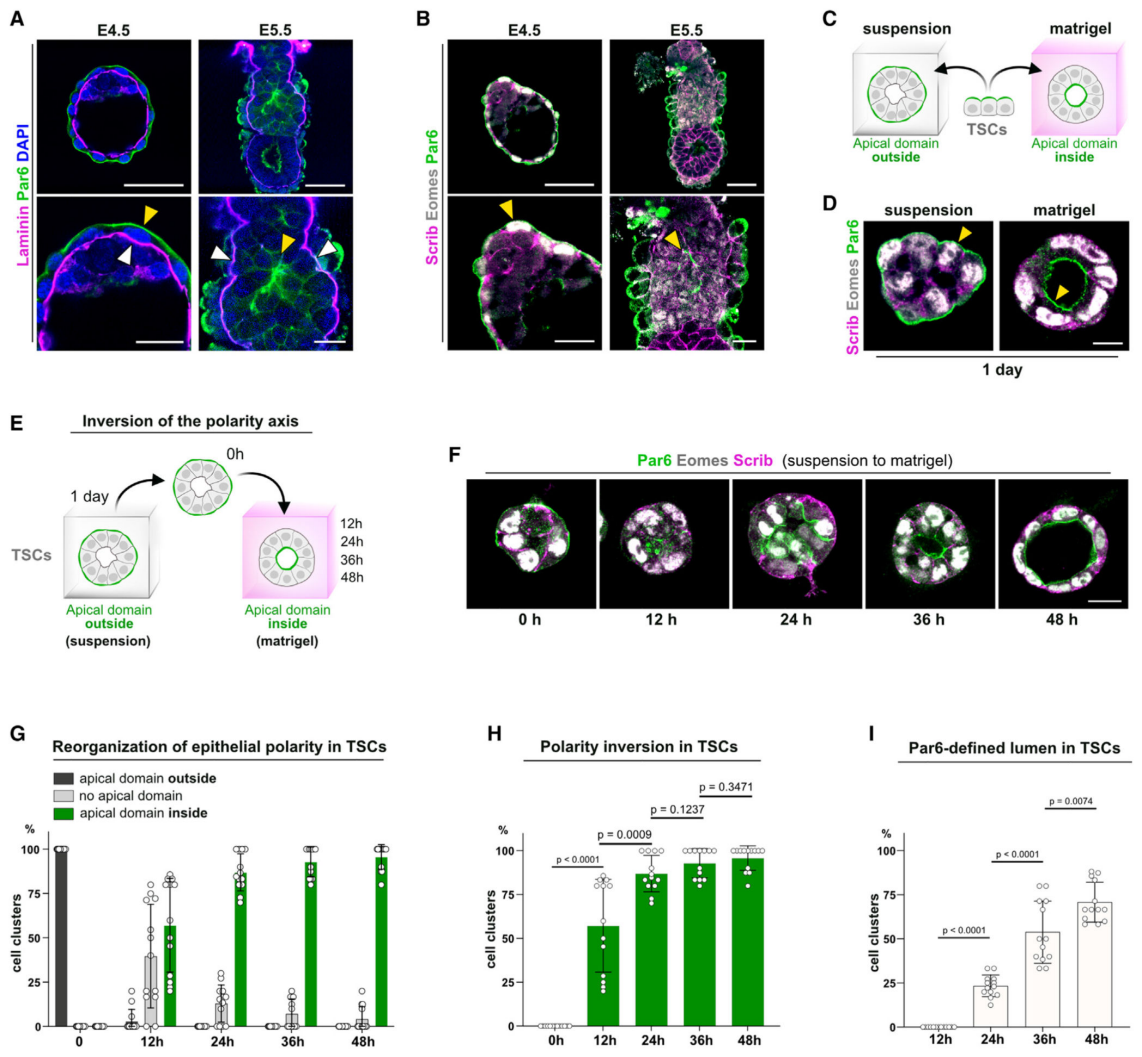
Inversion of the apical-basal polarity axis (A) Representative images of E4.5 and E5.5 embryos stained for Par6, laminin, and DAPI. Yellow arrowheads indicate the apical domain, white arrowheads indicate the BM. (B) Representative images of E4.5 and E5.5 embryos stained for Eomes, Par6, and Scrib. Arrowheads indicate the apical domain. (C) Schematic representation of TSCs cultured in suspension or in Matrigel for 24 h. (D) Representative images of TSCs stained for the Eomes, Par6, and Scrib after 1 day of culture in suspension or in Matrigel. Arrowheads indicate the apical domain. (E) Schematic representation of the TSC-based model of the polar TE to ExE transition. (F) Representative images of TSCs cultured in suspension for 1 day and then embedded in Matrigel. The cells were stained for the Eomes, Par6, and Scrib at 0 h, 12 h, 24 h, 36 h, or 48 h of culture in Matrigel. (G) Positioning of the apical domain in TSCs based on Par6 expression, n = 13 independent experiments for each time point; data represent mean ± SD. (H) Quantification of the TSC clusters exhibiting inversion of polarity, n = 13 independent experiments for each time point; data represent mean ± SD, two-tailed unpaired Student’s t test. (I) Quantification of lumen formation, defined by Par6 signal, n = 13 independent experiments for each time point; data represent mean ± SD, two-tailed unpaired Student’s t test. Scale bars (A) and (B), 50 μm (top) and 20 μm (bottom); (D) and (F), 10 μm. See also Figure S3.

Next, we sought to identify the cellular mechanism that reorganizes the epithelial polarity in the polar TE/ExE. In contrast with the pre-implantation epiblast, which consists of non-polarized cells that establish epithelial polarity de novo during the E4.5–E5.5 transition,^23,24^ the TE is already a polarized monolayer before implantation. The polar TE cells are positioned over the BM (Figure 4A, white arrowhead) and have defined basolateral (Scrib-positive) and apical (Par6-positive) membrane domains (Figures 4A and 4B). At E5.5, the BM of the visceral endoderm (VE) surrounds the trophoblast cells, which position their apical domains within the core of the ExE (Figure 4A). Staining for ECM proteins revealed the expression of laminin, collagen IV, and fibronectin at the BM, while the active form of integrin beta-1 was localized at the basal domain of the trophoblast cells contacting the BM (Figures 4A, S3A, and S3B). As the TSCs represent the multipotent trophoblast *in vitro*,^3^ we asked whether these cells can capture the epithelial reorganization of the polar TE/ExE in a controlled environment.

First, we aimed to establish a reductionist approach for modeling the spatial organization of the TE and ExE cells *in vitro* using TSCs. As the blastocyst is a free-floating structure, we placed the TSCs on a low attachment plate (suspension culture), where they formed cell clusters with apical membrane domains facing the outside environment. To model the surrounding BM in the post-implantation embryo, we embedded the TSCs into a hydrogel of ECM proteins (Matrigel), which resulted in the establishment of an apical domain within the cell cluster (Figures 4C and 4D). Both the TSCs cultured in suspension or in Matrigel exhibited no signs of differentiation and maintained the expression of the stem cell markers Cdx2, Gata3, Eomes, and Elf5^25^ (Figures 4D, S3C, and S3D).

Next, to mimic the E4.5 TE to E5.5 ExE transition, we cultured the TSCs for 1 day on a cell-repellent plate, and then we transferred the cell clusters into Matrigel for an additional culture of 12–48 h (Figure 4E). We found that the TSCs reorganized their epithelial polarity within 24 h of culture (Figures 4F, 4G, and S3C), corresponding with the 24-h period of the E4.5 to E5.5 transition *in vivo.* The TSCs inverted their apical-basal polarity axis, establishing apical domains inside the cell cluster (Figure 4H), which was associated with the formation of a central lumen, surrounded by Par6/Pdx-positive cell membranes (Figures 4F, 4I, and S3C).

**Figure 5:**
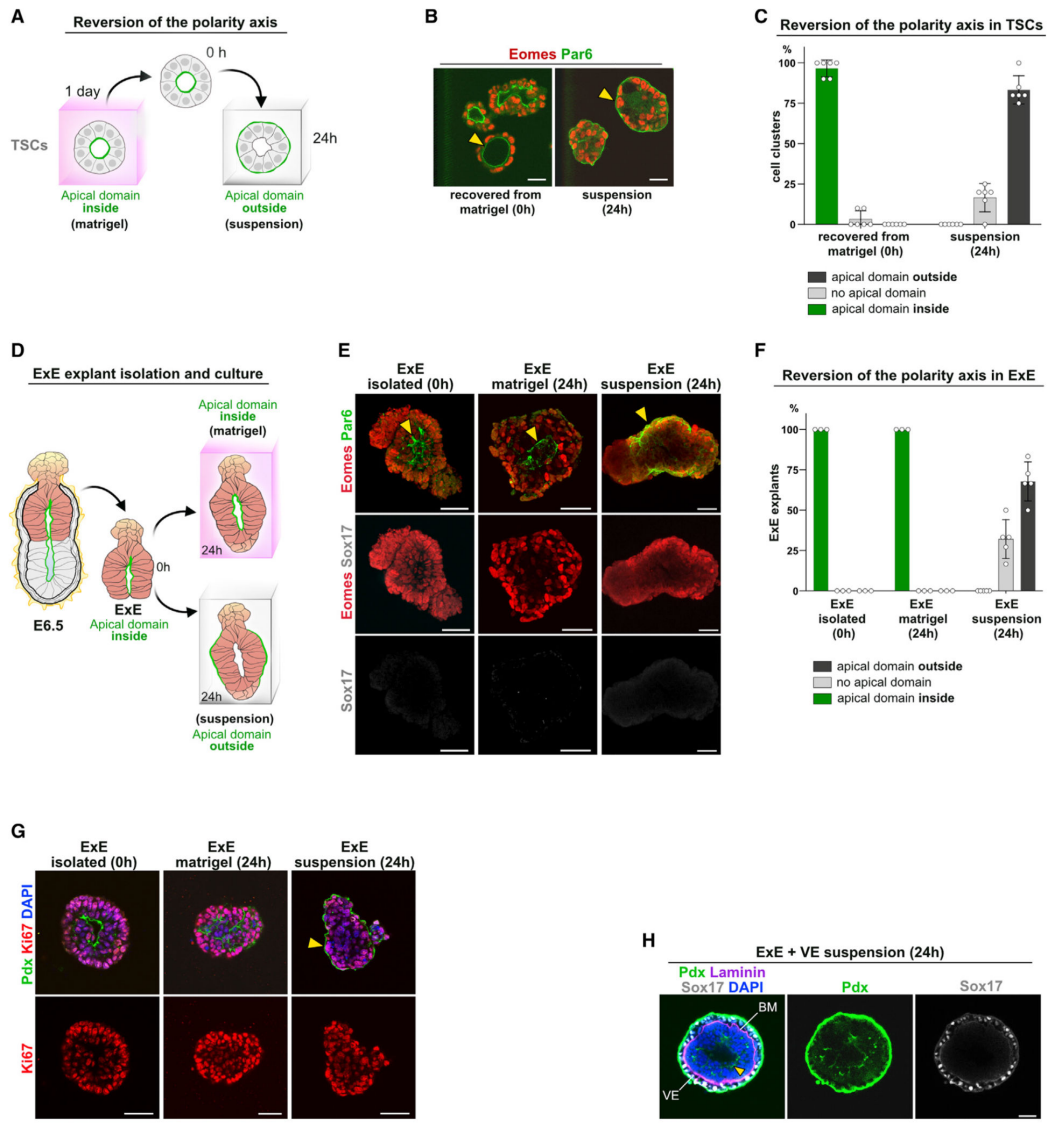
Reversion of the apical-basal polarity axis (A) Schematic representation of recovering the TSCs from Matrigel after 1 day of culture and subsequent culture in suspension for 24 h. (B) Representative images of TSCs cultured in Matrigel for 1 day were recovered and then cultured in suspension for 24 h. The cells were stained for Eomes and Par6. Arrowheads indicate the apical domain. (C) Quantification of the apical domain positioning in TSCs after the recovery from Matrigel (0 h) and after culture in suspension for 24 h, n = 6 independent experiments for each group. Data represent mean ± SD. (D) Schematic representation of the isolation and culture of ExE explants. (E) Representative images of ExE explants immediately after recovery (0 h) orcultured in Matrigel or suspension for 24 h were stained for Eomes, Par6, and the VE marker Sox17. (F) Quantification of the apical domain positioning of ExE explants; 0 h after recovery, n = 3; 24 h in Matrigel, n = 3; 24 h in suspension, n = 5 independent experiments. Data represent mean ± SD. (G) Representative images of ExE explants immediately after recovery (0 h) or cultured in Matrigel or suspension for 24 h were stained for Ki67, Pdx, and DAPI. Arrowhead indicates the apical domain, three independent experiments. (H) Representative images of ExE with intact VE cultured in suspension for 24 h and stained for Pdx, Laminin, Sox17, and DAPI. Arrowhead indicates the apical domain, three independent experiments. Scale bars (B), 30 μm; (E), (G), and (H), 50 μm.

To understand whether the inversion of the polarity axis is a reversible process, we cultured TSCs in Matrigel for 1 day, enabling the establishment of an apical domain inside the cell clusters. After that, we dissolved the Matrigel and transferred the TSC clusters on low attachment plates, where we cultured the cells in suspension for an additional 24 h (Figure 5A). We found that the TSCs established an apical domain facing the outside environment, indicating that the apical-basal polarity axis in these cells was reverted (Figures 5A–5C).

To examine whether the trophoblast cells of the embryo are also responsive to modulations in the external polarization cues, we isolated ExE explants by removing the VE and the epiblast. As this procedure requires the manual dissection of early embryos, we used E6.5 egg cylinders, which are larger than E5.5 concepti, enabling precise removal of the surrounding tissues (Figure 5D). We cultured the ExE explants embedded in Matrigel or in suspension for 24 h and found that the apical domain of the trophoblast cells was positioned within the explant (Matrigel) or predominantly repositioned to the external environment (suspension), respectively (Figures 5D–5F). Staining for the proliferation marker Ki67 confirmed the viability of the explants (Figure 5G). In contrast, explants with an intact overlaying VE cultured in suspension sustained the apical domain within the core of the ExE (Figure 5H).

Altogether, this shows that the trophoblast cells with stem cell properties are particularly responsive to polarization cues, which direct the orientation of the apical-basal polarity axis and, thereby, organize the tissue-scale architecture of trophoblast.

### Beta-1, -3, and -5 integrins transmit polarization cues, directing the orientation of the apical-basal axis

**Figure 6:**
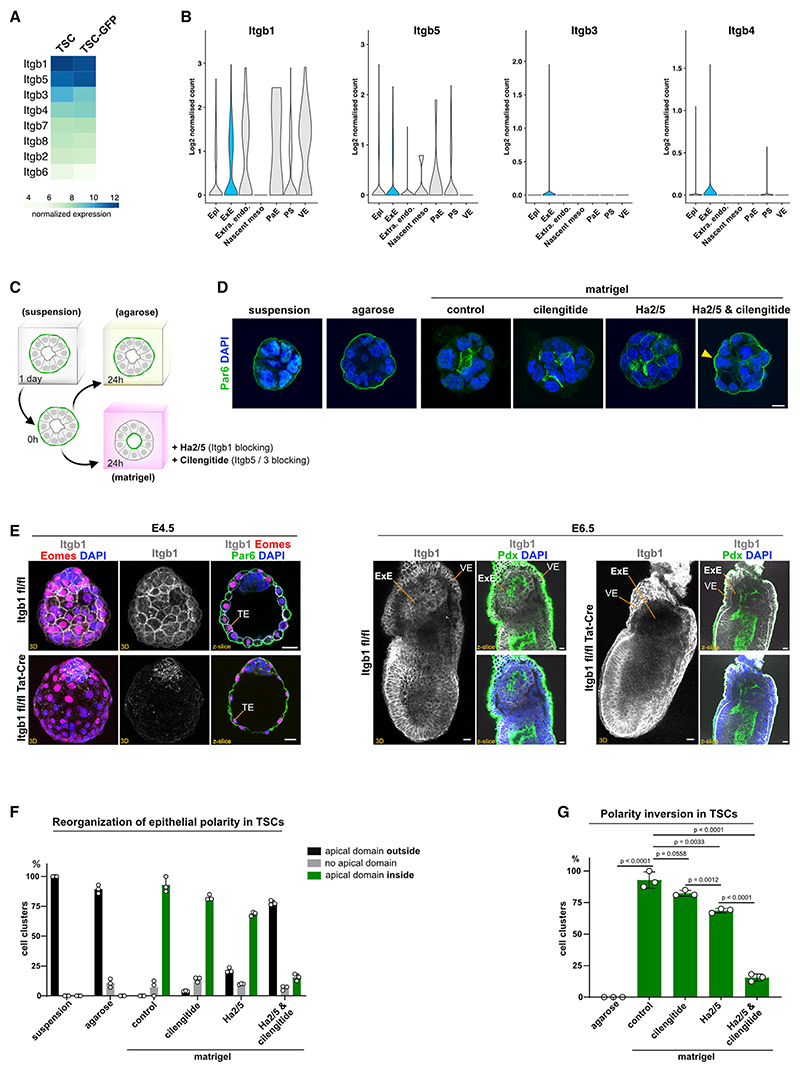
Polarization cues transmitted by beta-1, -3, and -5 integrins direct the orientation of the apical-basal axis (A) Heatmap plot of normalized expression levels of integrin beta subunits in TSCs, based on the microarray dataset of Adachi et al. (2013).^26^ (B) Violin plots of single-cell RNA sequencing (scRNA-seq) expression level of integrin beta subunits in E6.5 embryos, based on the scRNA-seq dataset of Pijuan-Sala et al. (2019).^27^ (C) Schematic representation of TSCs cultured in agarose or Matrigel after 1 day of culture in suspension. (D) Representative images of TSCs recovered from suspension and cultured in agarose or Matrigel for 24 h and stained for Par6, active integrin beta-1, and DAPI. The TSC medium used for the Matrigel culture was supplemented with cilengitide, Ha2/5, or a combination of cilengitide and Ha2/5. The control cells in Matrigel were cultured in TSC medium without cilengitide or Ha2/5. The apical domain in cilengitide and Ha2/5 treated TSCs is indicated with an arrowhead. (E) Representative images of non-treated (n = 6) and Tat-Cre-treated (n = 10) integrin beta-1 floxed embryos stained for apical polarity marker Par6 or Pdx, integrin beta-1, and DAPI, three independent experiments. (F) Quantification of the apical domain positioning in TSCs, n = 3 independent experiments for each group. Data represent mean ± SD. (G) Quantification of the TSC clusters exhibiting inversion of polarity, n = 3 independent experiments for each group. Data represent mean ± SD, two-tailed unpaired Student’s t test. Scale bars (D), 10 μm; (E), 20 μm (left, E4.5); 50 μm (right, E6.5) and 20 μm (high magnification). See also Figure S4.

To identify the receptors transmitting the polarization cues, we examined the expression of integrins in TSCs and the ExE. We used available transcriptomics datasets of two TSC lines (TSC and TSC-GFP),^26^ which revealed the presence of an array of alpha and beta integrin subunits in TSCs (Figures 6A and S4A). Among the beta subunits, beta-1 and beta-5 were predominantly expressed in both TSC lines (Figure 6A). Moreover, analysis of available single-cell RNA sequencing data^27^ showed that beta-1 and beta-5 integrin expression is also enriched in the ExE (Figures 6B, S4B, and S4C) and, similar to in the TSCs, beta-3 and low levels of beta-4 integrins were also detectable (Figures 6A and 6B).

Finally, we examined the functional significance of the integrin receptors in the reorganization of the polarity axis. After 1 day of culture in suspension, we embedded the TSC clusters into agarose, which lacks ECM proteins and, therefore, does not provide polarization cues. Accordingly, the direction of the apical-basal polarity axis remained unchanged in comparison with the initial culture in suspension (Figures 6C, 6D, 6F, and 6G). Alongside, we embedded TSCs in Matrigel (control), which resulted in polarity axis inversion. Interestingly, treatment with an integrin beta-1 blocking antibody (Ha2/5)^28^ did not block the polarity inversion effectively (Figures 6D–6G). To examine the role of integrin beta-1 *in vivo,* we implemented the *Tat-Cre/loxP* recombination approach for the deletion of conditional alleles in the trophoblast.^20^ Tat-Cre treatment of E3.5 embryos harboring integrin beta-1 floxed alleles^29^ resulted in a TE-specific depletion of the integrin beta-1 receptor by E4.5 (Figure 6E). After embryo transfer into surrogate mothers, both the non-treated (control) and the Tat-Cre-treated embryos established epithelial polarity in the ExE (Figure 6E), in accord with the results of the integrin beta-1 blocking in TSCs, *in vitro.* In addition, 3D culture of TSCs in the presence of cilengitide, a cyclic RGD pentapeptide that prevents the activation of beta-5 and beta-3 integrins,^30^ also did not fully abolish the reorganization of epithelial polarity (Figures 6D–6G). Only a combined treatment consisting of both Ha2/5 and cilengitide efficiently suppressed the inversion of the apical-basal polarity axis (Figures 6D–6G), showing that the integrin receptors act in concert and exhibit functional redundancy in transmitting the polarization cues that direct the orientation of the polarity axis.

## Discussion

The establishment of epithelial polarity during the pre-implantation development determines the cell fate specification of the TE, which has two major functions—to mediate the process of implantation and to give rise to the tissues of the placenta. This bifurcation of the TE functions becomes apparent during periimplantation embryogenesis and is associated with a reorganization of the tissue-scale architecture in the trophoblast.

**Figure 7:**
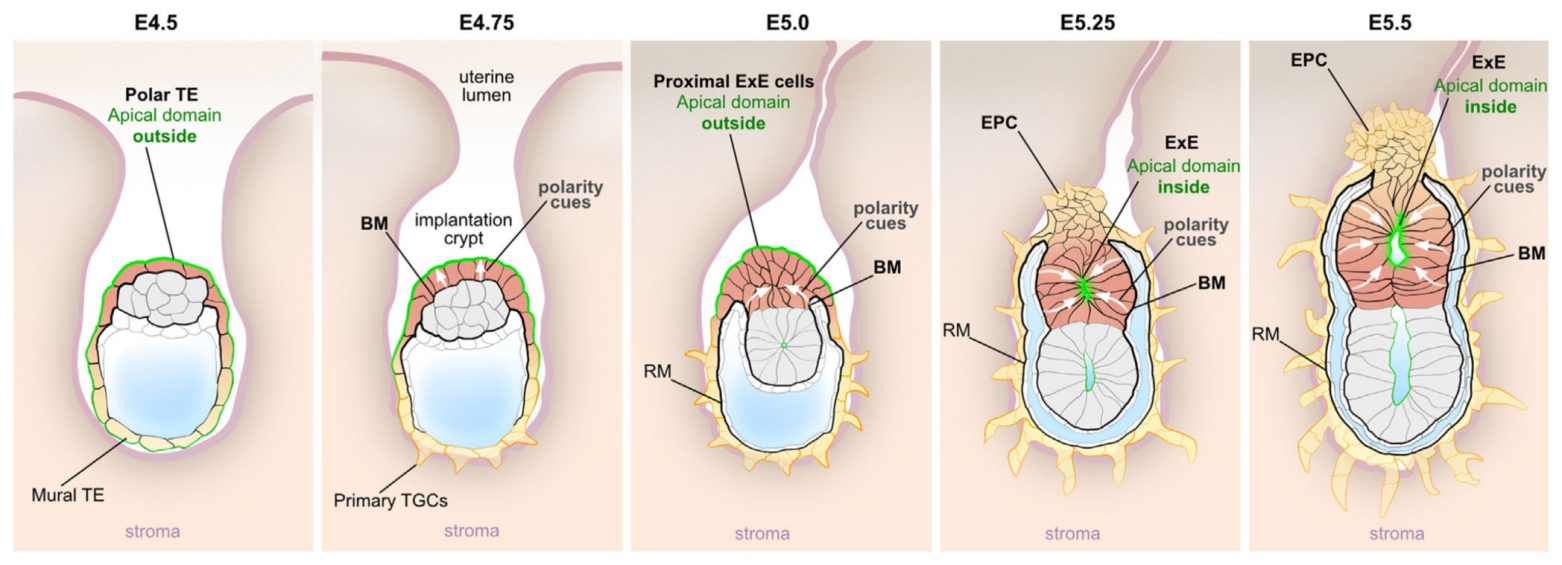
Polarity inversion reorganizes the stem cell compartment of the trophoblast lineage See the discussion for details.

At the blastocyst stage, the trophoblast is organized as an epithelial monolayer (Figure 7). Upon attachment to the uterine wall (E4.5–E4.75), the distal portion of the TE (mural TE) differentiates into TGCs, which lose their epithelial phenotype. At E5.0 only the most proximal cells of the ExE still face the outside environment, exhibiting their apical membrane domains toward the open space of the implantation crypt. This is where the second site of direct embryo-maternal interactions is formed between E5.0 and E5.5, as the proximal cells of the ExE give rise to the EPC. Thus, to establish the early embryo-maternal interface, the trophoblast cells undergo differentiation, which is associated with loss of epithelial morphology.

The trophoblast cells with stem cell properties, including the TSCs *in vitro,* remain epithelial. The polar TE transforms into ExE during the E4.5 to E5.5 transition via two concurrent processes—cell proliferation and inversion of the apical-basal polarity axis. The rapid increase in cell mass is initiated upon implantation, transforming the E4.5 polar TE monolayer into a nascent ExE at E5.0, which further expands in the proximal (EPC) and distal (ExE) directions between E5.0 and E5.5.

The reorganization of the epithelial polarity during the polar TE/ExE is directed by interactions between the BM and integrins. The BM of the VE surrounds the nascent E5.0 ExE, which, together with the epiblast, expand into the blastocoel cavity. At E5.0, only the proximal trophoblast cells exhibit an apical membrane, which faces the outside environment, whereas the inner trophoblast cells lack a defined apical domain. By E5.5, the proximal cells give rise to the EPC, while polarization cues from the BM, transmitted via integrin receptors, direct the formation of an inside apical domain in the ExE (Figure 7). On a tissue scale level, this results in the global inversion of the direction of the apical domain in the stem cell compartment of the trophoblast lineage between E4.5 (polar TE/outside) and E5.5 (ExE/inside). This process can be faithfully recapitulated *in vitro*, using TSCs that express the same set of integrin receptors.

The establishment of epithelial polarity in the pluripotent lineage also depends on polarization cues provided by the BM and transmitted via integrin receptors.^11,31,32^ At E4.5, the pre-implantation epiblast is a simple ball of non-polarized cells, which express the pluripotency transcription factors (TFs) Oct4 and Sox2 together with a set of so-called ancillary TFs, such as Nanog and Esrrb.^33^ During the peri-implantation stages, Oct4 relocates its genome occupancy, while the expression of the ancillary TFs is downregulated.^33,34^ This transcriptional rewiring is a hallmark of the transition from a pre-implantation (naive) to the early post-implantation (formative) states of pluripotency, which is associated with the initiation of morphogenesis and de novo establishment of epithelial polarity in the epiblast.^23,24^ Thus, as a result of concurrent morphogenetic processes, by E5.5 both the epiblast and the ExE are organized as polarized epithelia with apical domains facing the central cavity in the core of the egg cylinder (Figure 7).

The BM scaffolding surrounding the ExE constitutes primarily of laminins, collagen type IV, and nidogen-1.^16,35^ Genetic ablation of the laminin γ1 subunit results in peri-implantation lethality caused by failure of the BM assembly.^36^ Accordingly, it has been reported that laminin is a major component of the ECM niche that supports the proliferation of the ExE.^37^ In contrast, the deletion of nidogen-1 or collagen IV does not affect the early embryogenesis,^38–40^ indicating that laminin is the most critical component for BM formation at this developmental stage. Similarly, fibronectin knock-out embryos do not exhibit developmental defects before gastrulation,^41,42^ although *in vitro* studies suggest that fibronectin-integrin alpha-5 beta-1 interaction is involved in blastocyst attachment, while alpha-IIb beta-3 and alpha-V beta-3 integrins, which also bind fibronectin, are involved in the cell migration of the differentiating mural TE/TGCs.^43^

The recent study by Kim et al.^44^ showed that cells of the pre-implantation embryo are also sensitive to ECM-integrin signaling, which can provide inside positional cues to the blastomeres of the morula stage embryo, promoting their ICM fate. Although integrin beta-1 function is dispensable for the specification of the ICM,^44,45^ the authors found that integrin beta-1 activity is involved in the epiblast/PE patterning during blastocyst maturation and is required for the proper epithelial organization of the PE layer at E4.0.^44^

The process of epithelial polarity inversion has been well described *in vitro,* in epithelial cell lines such as the Madin-Darbey canine kidney (MDCK) cells. In suspension, the MDCK cells form cell clusters with apical membranes facing outward. Upon exposure to ECM (e.g., collagen gel), the MDCK cells invert their epithelial polarity axis and form a cyst with an inward apical surface.^46^ The reorientation of the polarity axis in MDCK cells depends on activation of the integrin beta-1, and this process is inhibited upon treatment with an integrin beta-1-blocking antibody.^47^ As trophoblast cells express several members of the beta integrin family, blocking the activity of integrin beta-1 is compensated by beta-5 and beta-3 integrins.

In MDCK cells, integrin activation induces Rac1, which promotes laminin deposition and BM assembly.^46,47^ *In vivo,* the BM is provided by the VE; thus, Rac1 function is likely dispensable for the polar TE/ExE transition. Accordingly, Rac1 ko embryos exhibit defects in cell migration at gastrulation,^48^ while conditional ablation of Rac1 in the VE diminishes the collective migration of the anterior visceral endoderm.^49^

The activation of integrins in MDCK cells also triggers a molecular switch that reorganizes the polarity axis when the cells are embedded in Matrigel. The polarization cues from the ECM, which are transmitted via the integrin beta-1/focal adhesion kinase (FAK)/p190RhoGAP complex suppress the RhoA/ROCK/Ezrin cascade at the apical domain that is initially facing the ECM. This promotes the transcytosis of apical proteins such as Ezrin and Pdx and their delivery to the newly formed apical domain in the center of the cell clump.^50^ *In vivo,* FAK is dispensable for peri-implantation embryogenesis, as FAK knock-out embryos exhibit defects at E8.0–E8.5,^51^ whereas loss of Pdx is not associated with embryonic lethality.^52^ Thus, similar to the functional redundancy of the integrin family, multiple factors and compensatory signaling circuits may act in concert, enabling the reorganization of the polarity axis in the context of the developing embryo.

In summary, we found that inversion of the polarity axis reorganizes the epithelial architecture of the TSC compartment during peri-implantation embryogenesis. While the inversion of the apical-basal axis has been extensively studied in MDCK cells, our study provides evidence for the developmental significance of this process. Future research can reveal to what extent the molecular principles mediating the reorganization of the polarity axis are shared in the trophoblast and the conventional epithelial *in vitro* model systems and whether other mammalian species use this process during early embryonic development.

### Limitations of the study

Although the TSCs culture approach in suspension and Matrigel provides an accessible model of the trophoblast reorganization, this reductionist platform is not applicable to examine the long-term effects of disrupting epithelial morphogenesis in the placental tissues. Such studies may require a multiplex genetic ablation of factors, such as the integrin beta subunits, in a trophoblast-specific manner *in vivo*. As integrin beta-1 function is critical for epiblast development,^32,45^ the Tat-Cre/*oxP* recombination approach used for the deletion of conditional alleles in the trophoblast enabled bypassing the peri-implantation lethality of the conventional (zygotic) integrin beta-1 knock-out. A limiting factor of this experimental approach is the requirement of mouse strains harboring conditional alleles. Moreover, a prerequisite for examining the functional redundancy of the beta integrins in the trophoblast lineage is the generation of a mouse line that carries homozygous conditional alleles for beta-1, -3, and -5 integrins, which is currently not available.

## Star* Methods

Detailed methods are provided in the online version of this paper and include the following:

- KEY RESOURCES TABLE
- RESOURCE AVAILABILITY
  ○ Lead contact
  ○ Materials availability
  ○ Data and code availability
- EXPERIMENTAL MODEL AND SUBJECT DETAILS
  ○ Mice
  ○ Cell lines
- METHOD DETAILS
  ○ 3D TSC culture
  ○ Isolation of embryos
  ○ Immunofluorescent staining
  ○ Whole-mount immunofluorescent staining
  ○ Tissue clearing
  ○ Tat-Cre treatment
  ○ 3D embryo culture and time-lapse microscopy
  ○ Transmission electron microscopy
- QUANTIFICATION AND STATISTICAL ANALYSIS
  ○ Microscopy and image analysis
  ○ Statistical analysis

## Star* Methods

### Key Resources Table

**Table:** 

REAGENT or RESOURCE	SOURCE	IDENTIFIER
Antibodies		
Mouse monoclonal anti-PARD6B (B-10)	Santa Cruz	Cat# sc-166405, RRID: AB_2267890
Rabbit polyclonal anti-APPL1	Cell Signaling	Cat# 3276, RRID: AB_2258386
Rabbit anti-Eomes	Abcam	Cat# ab23345, RRID: AB_778267
Rat monoclonal anti-Podocalyxin	R&D Systems	Cat# MAB1556, RRID: AB_2166010
Rabbit anti-Laminin	Sigma-Aldrich	Cat# L9393, RRID: AB_477163
Goat anti-Sox17	R&D Systems	Cat# AF1924, RRID: AB_355060
Goat polyclonal anti-Scrib (C-20)	Santa Cruz	Cat# sc-11049, RRID: AB_2254275
Mouse monoclonal anti-Cdx2	BioGenex	Cat# AM392, RRID: AB_2650531
Rat anti-Troma1	Home-made (kind gift from Prof. Rolf Kemler)	N/A
Rabbit monoclonal anti-Gata-3	Cell Signaling	Cat# 5852, RRID: AB_10835690
Rabbit polyclonal anti-Elf5	Thermo Fisher	Cat# 720380, RRID: AB_2688046
Rabbit polyclonal anti-RFP	Rockland	Cat# 600-401-379, RRID: AB_2209751
Rabbit anti-Ki67	Abcam	Cat# ab15580, RRID: AB_443209
Rabbit anti-Collagen IV	Abcam	Cat# ab6586, RRID: AB_305584
Mouse anti-Fibronectin	BD Biosciences	Cat# 610077, RRID:AB_2105706
Rat anti-9EG7 (active beta1 integrin)	Home-made (kind gift from Prof. Dietmar Vestweber)	N/A
Alexa Flour 488 Donkey Anti-Rabbit IgG (H + L)	Thermo Fisher	Cat# A-21202, RRID: AB_141607
Alexa Flour 594 Donkey Anti-Rabbit IgG (H + L)	Thermo Fisher	Cat# A-21207, RRID: AB_141637
Alexa Flour 594 Donkey Anti-Mouse IgG (H + L)	Thermo Fisher	Cat# A-21203, RRID: AB_141633
Alexa Flour 647 Donkey Anti-Rat IgG (H + L)	Thermo Fisher	Cat# A-21247, RRID: AB_141778
Alexa Flour 647 Donkey Anti-Mouse IgG (H + L)	Thermo Fisher	Cat# A-31571, RRID: AB_162542
Alexa Flour 647 Donkey Anti-Goat IgG (H + L)	Thermo Fisher	Cat# A-21447, RRID: AB_141844
Hamster monoclonal anti-CD29-FITC	BD Biosciences	Cat# 561796, RRID: AB_10894590
Chemicals, peptides, and recombinant proteins		
DAPI	Carl Roth	Cat# 6335.1
Tat-Cre recombinant protein	Millipore	Cat# SCR508
Matrigel, growth factor reduced	Corning	Cat# 356238
Agarose, low melting	SERVA	Cat# 11408.01
Fetal Bovine Serum	Biochrome	Cat# S0615
DMEM high glucose	Sigma-Aldrich	Cat# D5671
L-Glutamine Sigma-Aldrich	Sigma-Aldrich	Cat# G7513
Sodium pyruvate	Sigma-Aldrich	Cat# S8636
Non-essential amino acids	Sigma-Aldrich	Cat# M7145
Penicillin-streptomycin	Sigma-Aldrich	Cat# P4333
Trypsin-EDTA (0.25%)	Sigma-Aldrich	Cat# 25200056
2-mercaptoethanol	Sigma-Aldrich	Cat# M7522
Human FGF4	PeproTech	Cat#100-31-25
Heparin sodium cell culture tested	Sigma-Aldrich	Cat# H3149
Mineral oil	Sigma-Aldrich	Cat# M7167
M2	Sigma-Aldrich	Cat# M7167
KSOM medium	Millipore	Cat# MR-020P-5F
Donkey serum	VMR	Cat# S2170
Tyrode’s solution	Sigma-Aldrich	Cat# T1788
Formaldehyde (w/v), methanol-free 16%	Thermo Fisher	Cat# 28908
Triton X-100	Sigma-Aldrich	Cat# T9284
PBS without Ca/Mg	Sigma-Aldrich	Cat# D8537
Glycine	Sigma-Aldrich	Cat#G7126
Tween 20	Sigma-Aldrich	Cat# P7949
DMSO	Carl Roth	Cat# A994.1
Benzyl Benzoate 99+%	Thermo-Fisher	Cat# 105860010
Benzyl Alcohol	Sigma-Aldrich	Cat# 24122-2.5L-M
Methanol anhydrous 99.8%	Sigma-Aldrich	Cat# 322415-100ML
Sodium deoxycholate	Sigma-Aldrich	Cat# 30970
DMEM F-12	Invitrogen	Cat# 21331-046
Heat-inactivated FCS	Invitrogen	Cat# 10828028
ITS-X	Invitrogen	Cat# 51500-056
b-estradiol	Sigma-Aldrich	Cat# E8875
Progesterone	Sigma-Aldrich	Cat# P0130
N-acetyl-L-cysteine	Sigma-Aldrich	Cat# A7250
Sodium bicarbonate	Sigma Aldrich	Cat# S5761
KSR	Invitrogen	Cat# 10828028
Cilengitide	InvivoChem	Cat# V2806
Dextran	MP Biomedicals	Cat# 205195
MMP liable peptides - for DexMa Hydrogel	GenScript	CGPQGIAGQGCR (provided as HCl salt, purity >95%, custom synthesized
CGRGDS - for DexMa Hydrogel	GenScript	custom synthesized
8 arm PEG -VS (40kDa)	NOF Corporation	Cat# HGEO-400GS
TG - RGD – for PEG Hydrogel	GL Biochem	H-NQEQVSPL-RGDSPG-NH2 – custom synthesis
Critical commercial assays		
μ-Slide 8 Well	Ibidi	Cat# 80826
96-well low attachment plate	Corning	Cat# CLS3474
4-well plate	Thermo Fischer	Cat# 176740
Deposited data		
Microarray dataset of expression level of integrin subunits in TSC	Adachi K. et al., 2013	GEO: GSE28455
scRNA-seq dataset of expression level of integrin subunits in E6.5 embryos	Pijuan-Sala et al., 2019^27^	https://www.ebi.ac.uk/arrayexpress/experiments/E-MTAB-6967/; E-MTAB-6967
Experimental models: Cell lines		
Mouse:TSC_WT_LacZ	kind gift from Prof. Hubert Schorle, University of Bonn	N/A
Experimental models: Organisms/strains		
Mouse: WT C57BL/6	Bred in house	N/A
Mouse: WT CD1	Bred in house	N/A
Mouse: WT B6C3F1	Bred in house	N/A
Mouse: Eomes-mTomato-H2B:GFP	Probst et al., 2017^17^	N/A
Mouse: Lifeact-GFP	Riedl et al., 2008^22^	N/A
Mouse: mT/mG	Muzumdar et al., 2007^54^	JAX:007576
Mouse: Rosa-nTom-nGFP	Prigge et al. 2013^19^	JAX:023035
Software and algorithms		
ImageJ	Schindelin et al., 2012^53^	https://fiji.sc/; RRID: SCR_003070
IMARIS (V9.5.1)	Oxford Instruments	RRID: SCR_007370
Graphpad Prism	GraphPad	http://www.graphpad.com/; RRID: SCR_002798
Zen	Zeiss	RRID: SCR_013672

### Resource Availability

#### Lead contact

Further information and requests for resources and reagents should be directed to the lead contact, Ivan Bedzhov (ivan.bedzhov@mpi-muenster.mpg.de).

#### Materials availability

This study did not generate new unique reagents.

#### Data and code availability

- Accession numbers of publicly available microarray and scRNA-seq data used in this paper are listed in the key resources table.
- This paper does not report original code.
- Any additional information required to reanalyse the data reported in this paper will be shared by the lead contact upon request.

### Experimental Model and Subject Details

#### Mice

The mice used in this study were 6 weeks to 40 weeks old. The animals were maintained under a 14-h light/10-h dark cycle with free access to food and water. Female mice were housed in groups of up to 4 per cage, and male stud mice were housed individually. Embryos for experiments were obtained from wild-type and transgenic strains from mattings using females with a natural ovulation cycle. The mouse strains used in the experiments are B6C3F1 [(C57BL/6J x C3H/HeJ) F1], CD1, Eomes-mTomato-H2B:GFP,^17^ Rosa-nTom/nGFP,^19^ mT/mG,^53^ Lifeact-GFP^22^ and integrin beta-1 floxed.^29^ Animal experiments and husbandry were performed according to the German Animal Welfare guidelines and approved by the Landesamt für Natur, Umwelt und Verbraucherschutz Nordrhein-Westfalen (State Agency for Nature, Environment and Consumer Protection of North Rhine-Westphalia).

#### Cell lines

TSCs were maintained on plates coated with mitotically inactivated mouse embryonic fibroblasts in DMEM medium supplemented with 20% FBS, 2 mM L-glutamine, 1 mM sodium pyruvate, 50 U/ml penicillin-streptomycin, 0.1 mM non-essential amino acids, 0.1 mM 2-mercaptoethnal, 25 ng/ml Fgf4 and 1 μg/ml heparin (TSC medium) at 37 °C, 5% CO_2_ atmosphere in air.

### Method Details

#### 3D TSC culture

The cells were dissociated using 0.05% trypsin-EDTA and pelleted by centrifugation. For Matrigel culture, the cells were washed with PBS and then resuspended in Matrigel (Corning, 356231) (1000 cells per 1 μl of Matrigel), 20 μl of the cell suspension was plated per single well of 8-well ibidi μ-plate (80826-90, Ibidi). The plate was placed in a cell culture incubator at 37 °C, 5% CO2 atmosphere in air for 10 min for Matrigel to solidify. After that, each well was filled with 300 μl of TSC medium and cultured at 37 °C, 5% CO2 atmosphere in air. For suspension culture, the cells were cultured on a 96-well low attachment plate (CLS3474, Corning), 2 × 103 cells per 200 μl of TSC medium in each well and kept at 37 °C, 5% CO2 atmosphere in air. After 24 h of suspension culture, the TSC clumps were collected, washed with PBS and then embedded in 20 μl of Matrigel or 20 μl agarose (11408.01, SERVA) and plated in 8-well ibidi μ-plates. After the gels solidified, each the wells were filled with TSC medium (control) or TSC medium supplemented with 10 μM of cilengitide (V2806, InvivoChem) or 0.5 μg/ml of Ha2/5 blocking antibody (561796, BD Sciences) or a combination of cilengitide and Ha2/5 antibody.

#### Isolation of embryos

Embryos were recovered from CD1 or B6C3F1 females mated with wild-type males or transgenic reporter males. The day of the vaginal plug was considered as E0.5. Pre-implantation embryos (E3.5 and E4.5) were flushed out of uteri using M2 medium (Sigma, M7167), and Zona pellucida was removed by brief exposure to the acidic Tyrode’s solution (Sigma, T1788). Peri- and post-implantation embryos (E4.75, E5.0, E5.25, E5.5, E6.0 and E6.5) were manually recovered from decidua following previously reported methodology.^54^ The ExE explants were cultured in TSC medium at 37 °C, 5% CO2 atmosphere in air.

#### Immunofluorescent staining

E4.5 and E4.75 embryos or TSCs were fixed with 4% PFA for 10 min, washed two times in 5% FCS in PBS (washing solution), then permeabilized for 5 min at room temperature using a permeabilization buffer containing 0.1 Mglycine, 0.3% Triton X-100 in PBS and after that washed twice. The samples were incubated with primary antibodies diluted in blocking buffer (10% FCS in PBS), overnight at 4 °C. After samples were washed three times, they were incubated overnight at 4 °C with secondary antibodies and DAPI diluted in blocking buffer. After washing three times, the embryos were mounted on a glass bottom plate in droplets of PBS and covered with mineral oil for imaging. The TSCs in suspension culture were transferred into 8-well ibidi μ-plates in washing solution for imaging, whereas the TSCs cultured in Matrigel or agarose were directly imaged in the 8-well ibidi μ-plates used for the 3D culture.

E5.0, E5.25, E5.5, E6.0 and E6.5 embryos or ExE explants were fixed with 4% PFA for 15 min, washed two times in 0.2% Triton X-100 in PBS at room temperature. The fixed samples (up to 5) were transferred to one well of 96-well plate and then incubated at room temperature for 1 h in the following buffers - buffer 1 (0.2% Triton X-100 and 20% DMSO in PBS), buffer 2 (0.1% Tween-20, 0.1% Triton X-100, 0.1% deoxycholate, 0.1% NP40 and 20% DMSO in PBS), buffer 3 (0.2% Triton X-100, 0.3 M glycine and 20% DMSO in PBS) and buffer 4 (0.2% Triton X-100, 10% DMSO and 6% donkey serum in PBS). After that, the samples were incubated with primary antibodies diluted in a buffer containing 5% DMSO and 3% donkey serum in PTwH (0.2% Tween-20 and 10 μg/ml heparin in PBS) for 1 h at room temperature. After washing three times with 0.2% Triton X-100 in PBS, the samples were incubated with secondary antibodies and DAPI diluted in a buffer containing 3% donkey serum in PTwH for 1 h at room temperature. The samples were then washed three times again and mounted on a glass bottom plate in droplets of PBS and covered with mineral oil for imaging.

The primary and secondary antibodies used for the immunofluorescent staining are: Pard6B1 (1:200, Santa Cruz Biotechnology, sc-166405), Ezrin (1:200, Cell Signalling, 3276S), Eomes (1:200, Abcam, AB23345), Integrin B1 (1:100, BD Sciences, 561796), , MPI-MB, Podocalyxin (1:300, R&D systems, MAB1556), Laminin (1:500, Sigma, L9393), Sox17 (1:200, R&D, AF1924), Scrib (1:200, Santa Cruz, sc-11049), Cdx2 (1:200, Biogenex, MU392A-5UC), Troma-1 (1:50, kind gift from Prof. Dr. Rolf Kemler), Gata3 (1:300, Cell Signalling, 5852), Elf5 (1:200, Thermo Fisher Scientific, 720380), Ki67 (1:200, Abcam, AB15580), Collagen IV (1:200, Abcam, AB6586), Fibronectin (1:200, BD Bioscience, 610077), 9EG7 (1:200, a kind gift from Prof. Dr. Dietmar Vestweber), Alexa Flour 488 Donkey Anti-Mouse (1:500, Thermo Fisher Scientific, A21202), Alexa Flour 488 Donkey Anti-Rabbit (1:500, Thermo Fisher Scientific, A21206), Alexa Flour 594 Donkey Anti-Rabbit (1:500, Thermo Fisher Scientific, A21207), Alexa Flour 594 Donkey Anti-Mouse (1:500, Thermo Fisher Scientific, A21203), Alexa Flour 647 Donkey Anti-Rat (1:500, Thermo Fisher Scientific, A21247), Alexa Flour 647 Donkey Anti-Mouse (1:500, Thermo Fisher Scientific, A31571), Alexa Flour 647 Donkey Anti-Goat (1:500, Thermo Fisher Scientific, A21447), DAPI (1:500, Carl Roth, 6335.1).

#### Whole-mount immunofluorescent staining

Uterine tissues containing E4.5, E4.75, E5.0, E5.25, E5.5, E6.0 and E6.5 embryos were fixed with 4% PFA for 1 h, washed two times in 0.2% Triton X-100 in PBS at room temperature. The samples were transferred into 15 ml falcon tubes and incubated for 24 h in each of the following buffers, on a rotatory shaker, at 37 °C – buffer 1 (0.2% Triton X-100 and 20% DMSO in PBS), buffer 2 (0.1% Tween-20, 0.1% Triton X-100, 0.1% deoxycholate, 0.1% NP40 and 20% DMSO in PBS), buffer 3 (0.2% Triton X-100, 0.3 M glycine and 20% DMSO in PBS) and buffer 4 (0.2% Triton X-100, 10% DMSO and 6% donkey serum in PBS). After that, the samples were incubated for 72 h at 37 °C with primary antibodies diluted in a buffer containing 5% DMSO and 3% donkey serum in PTwH (0.2% Tween-20 and 10 μg/ml heparin in PBS) After washing with 0.2% Triton X in PBS 5 times over the course of 24 h at room temperature, the samples were incubated for 72 h at 37 °C with secondary antibodies and DAPI diluted in a buffer containing 3% donkey serum in PTwH. The samples were then washed using 0.2% Triton X-100 in PBS 5 times over the course of 24 h at room temperature and finally washed two times with PBS before the tissue clearing procedure.

The primary and secondary antibodies used for the whole-mount immunofluorescent staining are: RFP (1:100, Biomol, 600-401-379), Podocalyxin (1:200, R&D systems, MAB1556), Alexa Flour 488 Donkey Anti-Rabbit (1:100, Thermo Fisher Scientific, A21206), Alexa Flour 647 Donkey Anti-Rat (1:200, Thermo Fisher Scientific, A21247), DAPI (1:200, Carl Roth, 6335.1).

#### Tissue clearing

After the whole-mount staining, the uterine tissues, containing early embryos, were dehydrated using sequential incubation with 50% v/v methanol, 70% v/v methanol, 95% v/v methanol and 100% (v/v) anhydrous methanol, and 100% (v/v) methanol for 1 h each at room temperature. The samples were then incubated in clearing solution containing a 1:1 ratio of 100% anhydrous methanol and BABB solution of 1:2 v/v benzyl alcohol and benzyl benzoate, overnight at room temperature. On the next day, the samples were incubated overnight in the BABB solution and then transferred into fresh BABB solution for imaging.

#### Tat-Cre treatment

Zona-free E3.5 Rosa-nTom/nGFP or integrin beta-1 floxed embryos were incubated with 0.75 μM of Tat-Cre (Millipore, SCR508) in pre-warmed KSOM medium (Millipore, MR-020P-5F) for 1 h at 37 °C, 5% CO2 atmosphere in air, following a previously reported methodology 26. After that, the embryos were washed with KSOM medium and cultured in a 4-well plate (176740, Thermo Fisher) in IVC1 or KSOM medium for 24 h, at 37 °C, 5% CO2 atmosphere in air. On the next day, the E4.5 integrin beta-1 floxed embryos were transferred into pseudopregnant females and isolated at E6.5.

#### 3D embryo culture and time-lapse microscopy

Rosa-nTom/nGFP and Life-Act GFP embryos were isolated at E3.5 stage and cultured in a 4-well plate (176740, Thermo Fisher) in IVC1 medium for 24 h, at 37 °C, 5% CO2 atmosphere in air. On the next day the embryos (up to 10) were transferred into a 10 μl volume of a PEG or DexMA hydrogel in a single well of an 8-well ibidi μ-plate (80826-90, Ibidi). The PEG and DexMA hydrogels were prepared as previously described.^9^ The embryos were carefully positioned and embedded inside the hydrogel by mouth pipetting. The embedded embryos were incubated for 10 min in a cell culture incubator at 37 °C, 5% CO2 atmosphere in air to solidify the hydrogel. After that, 200 μl of modified IVC2 medium was added to each well. After 24 h, the medium was exchanged with fresh IVC2 and time-lapse recording was performed using the Andor Dragonfly spinning disc confocal microscope.

The IVC1 and IVC2 media compositions were previously described.^9,11,55,56^ IVC1 medium consists of DMEM F-12 (21331-046, Invitrogen), supplemented with 20% heat-inactivated FCS (10828028, Invitrogen), 0.5x Pen (25 U/ml) / Strep (25 μg/ml) (P4333, Sigma), 2 mM L-glutamine (G7513, Sigma), 1x ITS-X (51500-056, Invitrogen), 8 nM b-estradiol (E8875, Sigma), 200 ng/ml Progesterone (P0130, Sigma) and 25 μM N-acetyl-L-cysteine (A7250, Sigma). The slightly modified IVC2 medium consists of DMEM (12800017, Thermo Fisher Scientific) supplemented with 1.0 g/l NaHCO3 (S5761, Sigma Aldrich) 5% heat-inactivated FCS (10828028, Invitrogen), 30% (vol/vol) KSR (10828028, Invitrogen), 0.5x Pen (25 U/ml) / Strep (25 μg/ml) (P4333, Sigma), 2 mM L-glutamine (G7513, Sigma), 1x ITS-X (51500-056, Invitrogen), 8 nM B-estradiol (E8875, Sigma), 200 ng/ml Progesterone (P0130, Sigma), 25 μM N-acetyl-L-cysteine (A7250, Sigma).

#### Transmission electron microscopy

Embryos were fixed in 2% PFA, 2% glutaraldehyde in 0.1M cacodylate buffer, pH 7.4. Samples were embedded in 2% LMP-agarose and postfixed in 1% osmium tetroxide, 1.5% potassium ferrocyanide in 0.1M cacodylate buffer. After that, the samples were dehydrated stepwise in ethanol, including *en bloc* 0.5% uranyl acetate staining during 70% ethanol. The last dehydration step was performed in propylene oxide followed by epon embedding. The samples were sectioned stepwise on an ultramicrotome (UC6, Leica) until the region of interest and 60-nm ultrathin sections were collected on a formvar-coated one-slot grid and counterstained with lead. The samples were imaged on a FEI-Tecnai 12-electron microscope at 80 kV (Thermo Fisher Scientific), and characteristic images were taken with a 2 K CCD-Veleta camera (EMSIS).

### Quantification and Statistical Analysis

#### Microscopy and image analysis

Imaging of the fixed samples was performed using Zeiss LSM 780 confocal microscope. For live imaging, Andor Dragonfly spinning disc confocal microscope equipped with a sCMOS camera was used. The samples were imaged in an incubation chamber providing stable culture conditions of 37 °C and 5% CO2 atmosphere in air. The live imagining was performed using 488 nm and 525 nm excitation lasers with minimum laser power (5-8% laser power) and images of 2048 x 2048 pixels were captured every 30 min, with an optical slice distance of 2 μm, using 20x CFI P-Apo Lambda objective.

#### Statistical analysis

All the experiments presented in this manuscript are reproduced at least in three independent experiments. Images are shown as the representative of all independent experiments. Statistical analyses were performed using GraphPad Prism 8.1. Values are presented as means ± SEM. Statistical significance (p value) was calculated using an unpaired Student’s t-test or one-way ANOVA.

## Supplementary Material

Supplementary Material

## Highlights

- Model describing trophoblast morphogenesis during the implantation stages
- Tissue-scale inversion of the epithelial polarity axis reorganizes the trophoblast
- Polarity inversion can be recapitulated *in vitro* using trophoblast stem cells
- Beta-1, -3, and -5 integrins co-operate to transmit polarization cues from the BM

## In brief

The tissues of the placenta originate from a small population of trophectodermal cells that undergo a fundamental morphogenetic transformation during the implantation stages. Ozguldez et al. found that this process is mediated by a tissue-scale inversion of the apical-basal polarity axis, reorganizing the stem cell compartment of the trophoblast lineage.
