# Supplementary material for: Polarity inversion reorganizes the stem cell compartment of the trophoblast lineage
Source: Cell Rep. Author manuscript; Available in PMC 2023 Sep 5. (PMC10157138; doi:10.1016/j.celrep.2023.112313)
Supplement: Supplementary Material [file EMS185283-supplement-Supplementary_Material.pdf]

Cell Reports, Volume *42*

Supplemental information

Polarity inversion reorganizes the stem cell compartment of the trophoblast lineage

Hatice O. Ozguldez, Niraimathi Govindasamy, Rui Fan, Hongyan Long, Karina Mildner, Dagmar Zeuschner, Britta Trappmann, Adrian Ranga, and Ivan Bedzhov

Figure S1

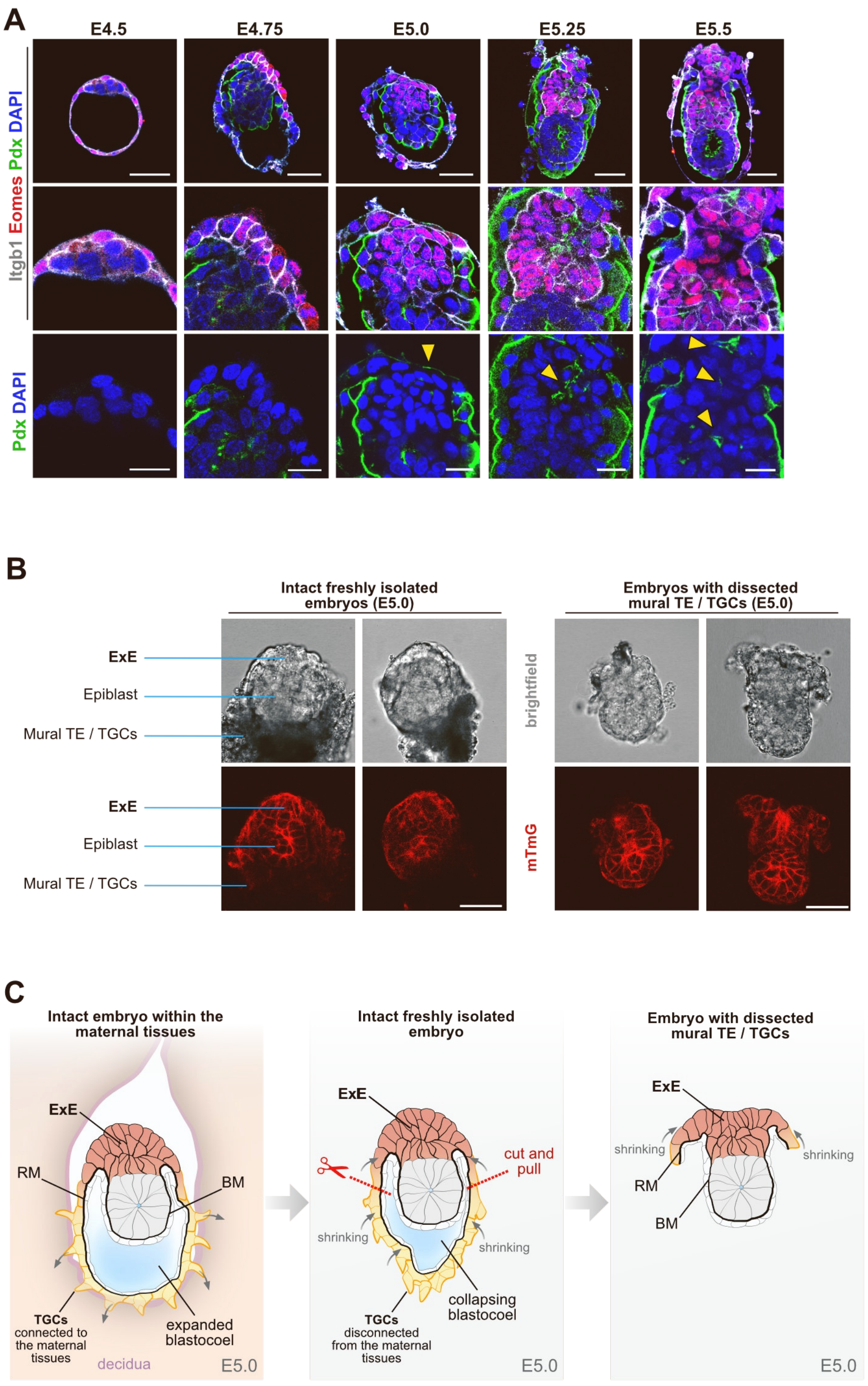

Figure S1. Reorganisation of epithelial polarity in the polar TE / ExE.
A) E4.5, E4.75, E5.0, E5.25 and E5.5 embryos stained for integrin beta-1, Eomes, Pdx and DAPI. Arrowheads indicate the apical domain.
B) Live E5.0 mT/mG embryos expressing membrane tdTomato.
C) Schematic representation of an intact E5.0 embryo within the implantation site, after isolation and following the mural TE / TGCs removal.
Scale bars A = 50 µm (top panel) and 20 µm (middle and bottom panels); B = 50 µm
Related to Figure 1.

Figure S2

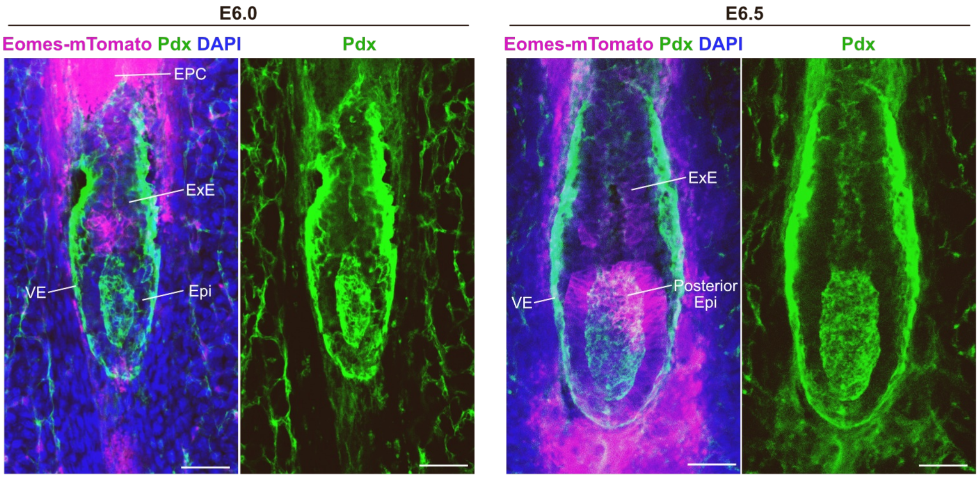

Figure S2. Trophoblast morphogenesis.
Whole-mount staining for mTom, Pdx and DAPI of uterine tissues containing E6.0 and E6.5 Eomes-mTom embryos.
At E6.5 Eomes is also expressed in the posterior epiblast.
Scale bars = 50 µm.
Related to Figure 2.

Figure S3

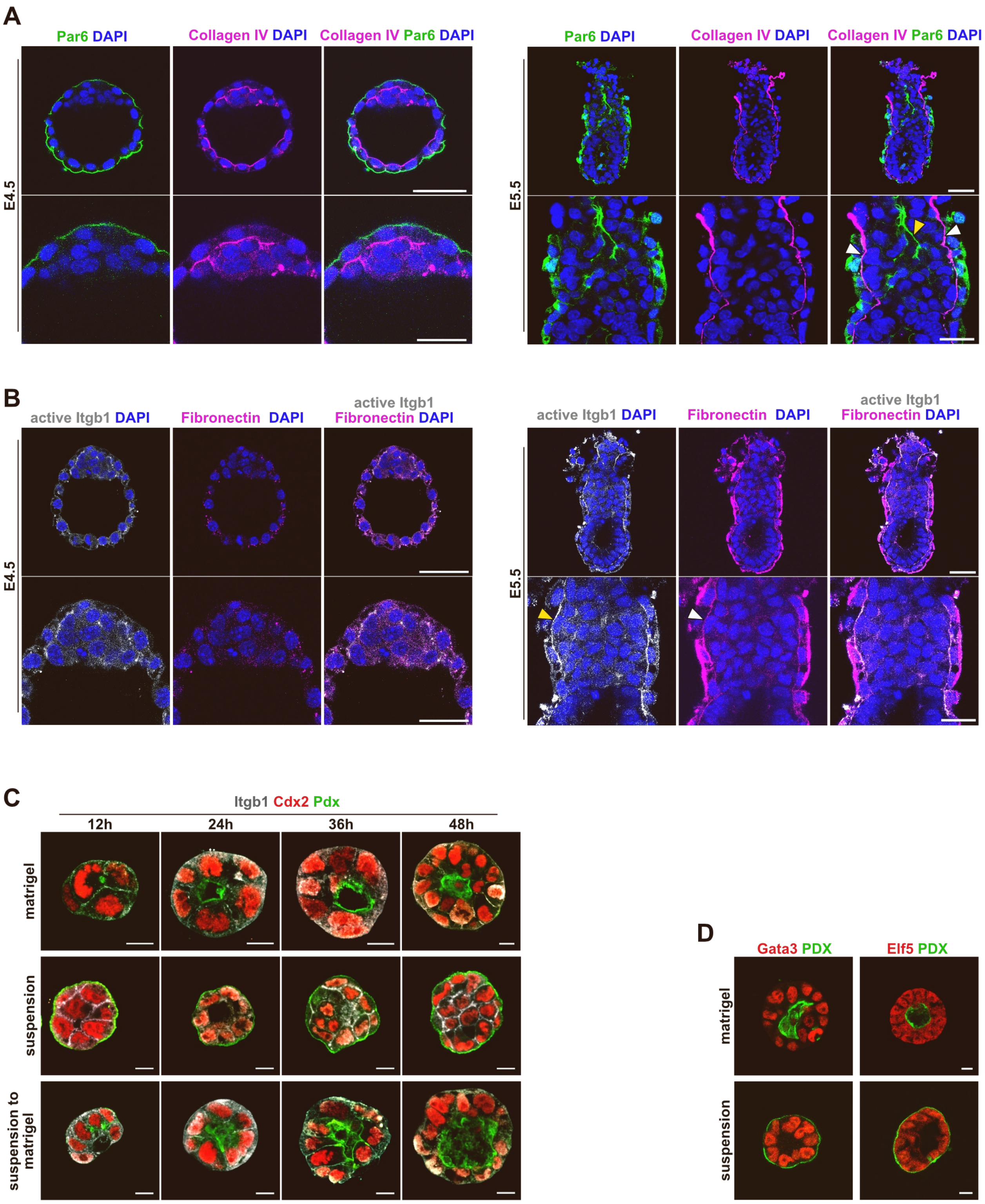

Figure S3. Inversion of the apical-basal polarity axis in TSCs.

A) E4.5 and E5.5 embryos stained for Par6, collagen IV and DAPI. Yellow arrowheads indicate the apical domain, white arrowheads indicate the BM.

B) E4.5 and E5.5 embryos stained for active integrin beta-1, fibronectin and DAPI. The yellow arrowhead indicates active integrin beta-1, white arrowhead indicates fibronectin.

C) TSCs cultured in Matrigel, in suspension or transferred from suspension to Matrigel and then cultured for 12 h, 24 h, 36 h or 48 h and stained for Cdx2, Pdx and integrin beta-1.

D) TSCs cultured in Matrigel and in suspension for 24 h and stained for Pdx, Gata3 or Elf5.

Scale bar A, B = 50 µm (top panels) and 25 µm (bottom panels); C, D = 10 µm

Related to Figure 4.

Figure S4

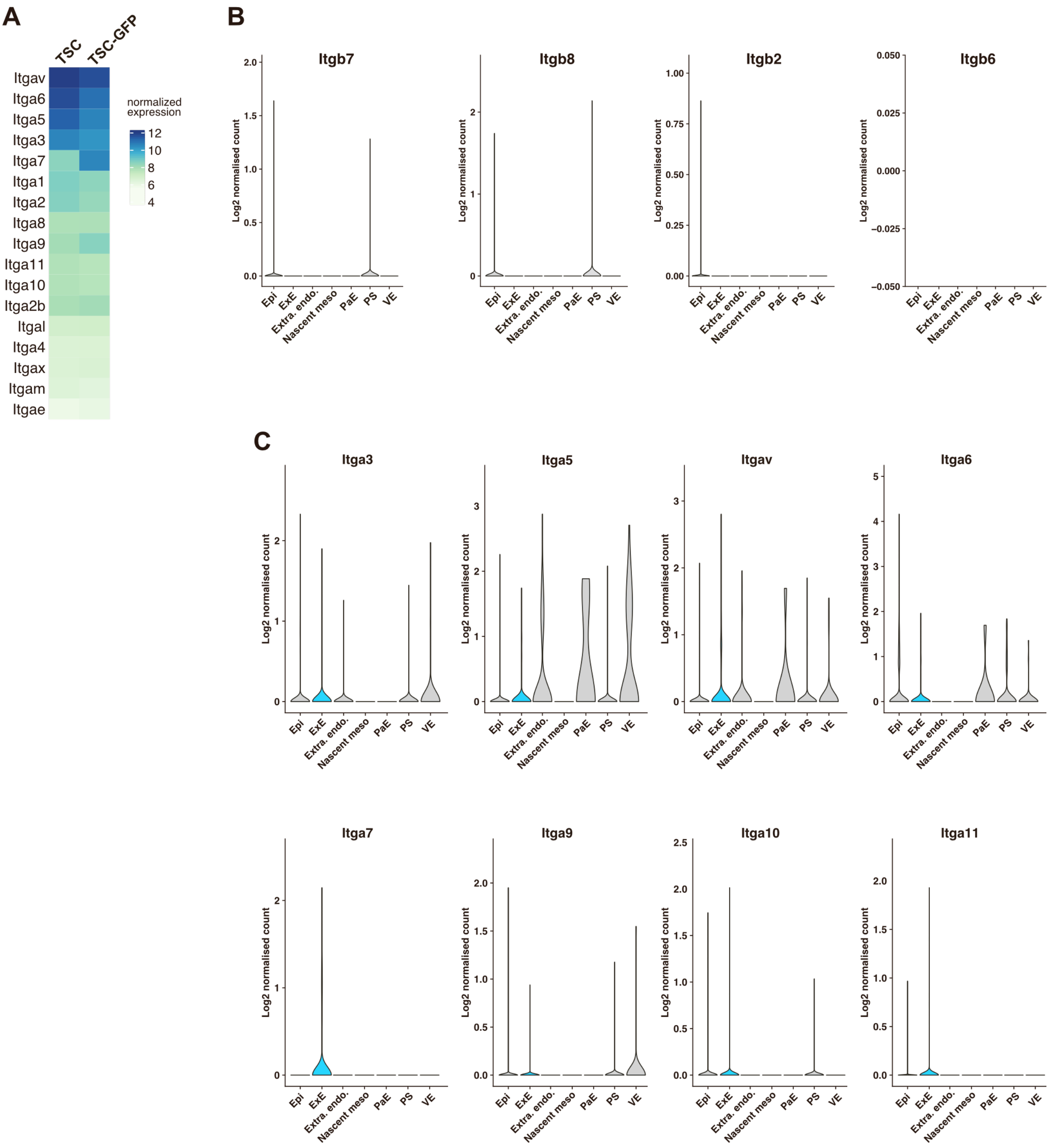

Figure S4. Expression of integrin subunits in TSCs and embryos.

A) Heatmap plot of normalised expression levels of integrin alpha subunits in TSCs, based on the microarray dataset of Adachi K. et al., 2013.

B) Violin plots of scRNA-seq expression level of integrin beta subunits in E6.5 embryos, based on the scRNA-seq dataset of Pijuan-Sala et al., 2019.

C) Violin plots of scRNA-seq expression level of integrin alpha subunits in E6.5 embryos, based on the scRNA-seq dataset of Pijuan-Sala et al., 2019.

Related to Figure 6.
